# Supplementary material for: Protocol for the economic evaluation of metacognitive therapy for cardiac rehabilitation participants with symptoms of anxiety and/or depression
Source: BMJ Open. 2020 Sep 10;10(9):e035552. doi: 10.1136/bmjopen-2019-035552 (PMC7485258; doi:10.1136/bmjopen-2019-035552)
Supplement: Supplementary data [file bmjopen-2019-035552supp001.pdf]

Protocol for the economic evaluation of metacognitive therapy for cardiac rehabilitation participants with symptoms of anxiety and/or depression

Correspondence to: gemma.shields@manchester.ac.uk

Supplementary material

Economic patient questionnaire

PATHWAY study

Funder Ref: *NIHR: RP-PG-1211-20011*

Participant ID: ☐☐☐

Participant initials: ☐☐☐

1. Use of hospital inpatient services

Date questionnaire completed ☐☐ DD ☐☐ MM ☐☐☐☐ YYYY

Please tell us about each **planned hospital overnight stay** you may have had **in the last 3 months** *(please do not include any hospital outpatient appointments, day hospital appointments or accident and emergency services here, we will ask you for information about these in questions 2, 3 and 4).*

a. Have you had any planned hospital overnight stays during the last 3 months?

Yes ☐ No ☐ Don't know ☐

b. **If YES**, please tell us about the department or specialty, the name of the hospital, your admission date and the number of days admitted in the box below. **Please record each admission on a separate line.**

Department – please give name/type of department or the reasons you were there.	Name of Hospital	Admission Date (MM/YYYY)	Number of inpatient days.	Don't know *(please tick if unsure how many days)*
e.g. Manchester Heart Centre	Manchester Royal Infirmary	12 / 2014	4 days	
		MM / YYYY	____ days	
		MM / YYYY	____ days	
		MM / YYYY	____ days	
		MM / YYYY	____ days	
		MM / YYYY	____ days	
		MM / YYYY	____ days	
		MM / YYYY	____ days	
		MM / YYYY	____ days	

PATHWAY EPQ Baseline v2 14JUN2016

1

Protocol for the economic evaluation of metacognitive therapy for cardiac rehabilitation participants with symptoms of anxiety and/or depression

Correspondence to: gemma.shields@manchester.ac.uk

PATHWAY study

Funder Ref: *NIHR: RP-PG-1211-20011*

Participant ID: ☐☐☐

Participant Initials: ☐☐☐

2. Use of hospital outpatient appointments (4 hours or less)

Please tell us about any **planned hospital outpatient** appointments (4 hours or less) in the last **3 months** *(please do not include any hospital inpatient admissions, day hospital appointments or accident and emergency services here, we ask you for information about these in questions 1, 3 and 4).*

If the participant has not used a service please Tick 'No'. If they don't know whether they have used a service, then please tick the 'Don't know' box. Thank you.

c. Have you attended any hospital outpatient appointments which lasted for **4 hours or less** during the last 3 **months**?

Yes ☐ No ☐ Don't know ☐

d. **If YES**, please tell us about the department or specialty and the number of appointments which **lasted 4 hours or less**.

**Department or specialty**	**Total number of visits during the last 3 months**	**Total number of visits during the last month**
Please list each type of department/clinic or specialty separately and tell us the number of visits for this department/clinic or specialty		

PATHWAY EPQ Baseline v2 14JUN2016

2

Protocol for the economic evaluation of metacognitive therapy for cardiac rehabilitation participants with symptoms of anxiety and/or depression

Correspondence to: gemma.shields@manchester.ac.uk

PATHWAY study

Funder Ref: *NIHR: RP-PG-1211-20011*

Participant ID: ☐☐☐

Participant initials: ☐☐☐

3.Use of day hospital appointments (lasting more than 4 hours)

Please tell us about any **planned day hospital appointments** (lasting more than 4 hours but not overnight) in the last 3 months *(please do not include any hospital inpatient or outpatient hospital appointments or accident and emergency services here, we ask you for information about these in questions 1, 2 and 4).*

If the participant has not used a service please Tick 'No'. If they don't know whether they have used a service, then please tick the **'Don't know'** box. Thank you.

a. Have you attended any planned day hospital appointments which lasted for **more than 4 hours (but not overnight)** during the last 3 months?

Yes ☐ No ☐ Don't know ☐

b. **If YES**, tell us about the department or specialty and the number of appointments **more than 4 hours (but not overnight)**.

**Department or specialty** *(e.g. minor surgery, dialysis, chemotherapy, other diagnostic procedures)*	**Total number of visits during the last 3 months**	**Total number of visits during the last month**
Please list each type of department/clinic or specialty separately and tell us the number of visits for this department/clinic or specialty		

PATHWAY EPQ Baseline v2 14JUN2016

3

Protocol for the economic evaluation of metacognitive therapy for cardiac rehabilitation participants with symptoms of anxiety and/or depression

Correspondence to: gemma.shields@manchester.ac.uk

PATHWAY study

Funder Ref: *NIHR: RP-PG-1211-20011*

Participant ID: ☐☐☐

Participant initials: ☐☐☐

4. Use of accident and emergency (A&E) services

Please tell us about any accident and emergency (A&E) service use in the last 3 months *(please do not include any planned hospital inpatient admissions, hospital outpatient or day hospital appointments here, we ask you for information about these in questions 1, 2 and 3).*

If the participant has not used a service please Tick 'No'. If they don't know whether they have used a service, then please tick the **'Don't know'** box. Thank you.

a. Have you attended an Accident and Emergency (A&E) unit during the last **3 months**?

Yes ☐ No ☐ Don't know ☐

b. **If yes**, please tell us about the number of A&E visits you had which **did not lead** to a hospital admission.

**Accident and Emergency visits not leading to inpatient admission**	
**Total number of visits during the last 3 months?**	**Total number of visits during the last month?**

c. Were you admitted into a hospital as an inpatient directly from the Accident and Emergency (A&E) unit during the last **3 months**?

Yes ☐ No ☐ Don't know ☐

d. **If yes**, please tell us about the number of A & E visits you had which **did lead to a** hospital admission. Please tell us about the Accident and Emergency visit in the table below and put details of the inpatient stay in the table in question 1. Thank you.

**Accident and Emergency visits leading to inpatient admission**	
**Total number of visits during the last 3 months?**	**Total number of visits during the last month?**

PATHWAY EPQ Baseline v2 14JUN2016

4

Protocol for the economic evaluation of metacognitive therapy for cardiac rehabilitation participants with symptoms of anxiety and/or depression

Correspondence to: gemma.shields@manchester.ac.uk

PATHWAY study

Funder Ref: *NIHR: RP-PG-1211-20011*

Participant ID: ☐☐☐

Participant initials: ☐☐☐

5. Use of primary and community based health services

Please tell us whether and how much you have used any of the following services in the **last 3 months** *(this section continues on the next page).*

If the participant has not used a service please enter '*0*'. If they don't know whether they have used a service, then please tick the '**Don't know**' box.

GP practice services	Total visits in the last 3 months?	Total visits in the last month?	Don't know
GP (at the surgery/practice)			
GP (at your home)			
Practice Nurse (at the surgery)			
Nurse (at your home)			
Community or primary care based cardiac unit			
Walk-in centre			
Counsellor or mental health worker			
Other *(please specify)*			
Other *(please specify)*			
Other *(please specify)*			

PATHWAY EPQ Baseline v2 14JUN2016

5

Protocol for the economic evaluation of metacognitive therapy for cardiac rehabilitation participants with symptoms of anxiety and/or depression

Correspondence to: gemma.shields@manchester.ac.uk

PATHWAY study

Funder Ref: *NIHR: RP-PG-1211-20011*

Participant ID: ☐☐☐

Participant initials: ☐☐☐

6. Social support services (accessed outside the hospital)

Please tell us whether and how much you have used any of the following social support services in the last **3 months**.

If the participant has not used a service please Tick 'No'. If they don't know whether they have used a service, then please tick the '**Don't know**' box.

Yes ☐ No ☐ Don't know ☐

**Other social support services** *(e.g. social worker, home help, care worker, occupational or physiotherapist) (please specify)*	**Total visits in the last 3 months?**	**Total visits in the last month?**	**Don't know**

PATHWAY EPQ Baseline v2 14JUN2016

6

Potential covariates for the trial analysis

- Age
- Gender
- Service use costs prior to baseline
- Baseline EQ-5D score

Protocol for the economic evaluation of metacognitive therapy for cardiac rehabilitation participants with symptoms of anxiety and/or depression
Correspondence to: gemma.shields@manchester.ac.uk

- Baseline HADs score
- Medication for depression and/or anxiety
- Site BMI
- Ethnicity
- Marital status
- Living arrangements
- Employment status
- Highest qualification
- Smoking status
- Alcohol units per month
- Presence of co-morbidities
- Previous cardiovascular event
- Age at first cardiovascular event
- Psychological therapies for anxiety or depression

Model sensitivity analysis

Assumptions/variables	Changes	Rationale
Time horizon	• Extension of the timeframe up to ten years and lifetime	A five-year time horizon has been chosen for the primary analysis of the economic model, to extend from the trial time horizon but with consideration of the lack of longer-term evidence for MCT. A time horizon equal to a patient's lifetime is typically preferred by NICE [12]. The use of this time horizon will be explored in sensitivity analysis and presented alongside discussion of the limitations.
Adherence to MCT	• Assuming a higher proportion of patients attend MCT sessions	Not everyone who is offered psychological therapy will attend, the trial data will allow us to see what proportion of participants who are offered MCT attend zero sessions. The model will look at the cost-effectiveness of MCT if attendance is improved.
Duration of MCT effect	• Exploring the assumed duration of MCT effect	The trial follow-up is 4 and 12-months, therefore we will be able to see whether MCT effect is sustained at 12-months (4-month signifies end of treatment). The model will explore the impact of different assumptions about the duration of MCT effect (e.g. how long effect is sustained and whether effect declines over time).
Repeated MCT intervention	• Addition of refresher sessions for participants who respond to MCT therapy	Evidence for the effectiveness of MCT is limited long time horizons. MCT requires participants to use the skills they learn during sessions to adapt their thoughts. Over time, there will be the potential for participants to forget the skills they learnt and therefore, refresher training sessions might be needed. The model will include a scenario to explore how repeated intervention might sustain treatment effect and the implantations of this for costs and QALYs.

Protocol for the economic evaluation of metacognitive therapy for cardiac rehabilitation participants with symptoms of anxiety and/or depression

Correspondence to: gemma.shields@manchester.ac.uk

MCT delivery	• Adjustments to the cost of MCT therapy	Assuming effectiveness of MCT remains unchanged, the model will explore how changes in delivery (e.g. staff delivering MCT) may impact cost-effectiveness.
